# Development of a gene signature associated with iron metabolism in lung adenocarcinoma

**DOI:** 10.1080/21655979.2021.1954840

**Published:** 2021-07-29

**Authors:** Junqi Qin, Zhanyu Xu, Kun Deng, Fanglu Qin, Jiangbo Wei, Liqiang Yuan, Yu Sun, Tiaozhan Zheng, Shikang Li

**Affiliations:** a Department of Thoracic and Cardiovascular Surgery, The First Affiliated Hospital of Guangxi Medical University, Nanning, Guangxi Zhuang Autonomous Region, P. R. China; b School of Information and Management, Guangxi Medical University, Nanning, Guangxi Zhuang Autonomous Region, P. R. China

**Keywords:** Lung adenocarcinoma, gene signature, risk score, survival, precise treatment

## Abstract

There are few studies on the role of iron metabolism genes in predicting the prognosis of lung adenocarcinoma (LUAD). Therefore, our research aims to screen key genes and to establish a prognostic signature that can predict the overall survival rate of lung adenocarcinoma patients. RNA-Seq data and corresponding clinical materials of 594 adenocarcinoma patients from The Cancer Genome Atlas(TCGA) were downloaded. GSE42127 of Gene Expression Omnibus (GEO) database was further verified. The multi-gene prognostic signature was constructed by the Cox regression model of the Least Absolute Shrinkage and Selection Operator (LASSO). We constructed a prediction signature with 12 genes (HAVCR1, SPN, GAPDH, ANGPTL4, PRSS3, KRT8, LDHA, HMMR, SLC2A1, CYP24A1, LOXL2, TIMP1), and patients were split into high and low-risk groups. The survival graph results revealed that the survival prognosis between the high and low-risk groups was significantly different (TCGA: P < 0.001, GEO: P = 0.001). Univariate and multivariate Cox regression analysis confirmed that the risk value is a predictor of patient OS (P < 0.001). The area under the time-dependent ROC curve (AUC) indicated that our signature had a relatively high true positive rate when predicting the 1-year, 3-year, and 5-year OS of the TCGA cohort, which was 0.735, 0.711, and 0.601, respectively. In addition, immune-related pathways were highlighted in the functional enrichment analysis. In conclusion, we developed and verified a 12-gene prognostic signature, which may be help predict the prognosis of lung adenocarcinoma and offer a variety of targeted options for the precise treatment of lung cancer.

## GRAPHICAL ABSTRACT

**Figure:**
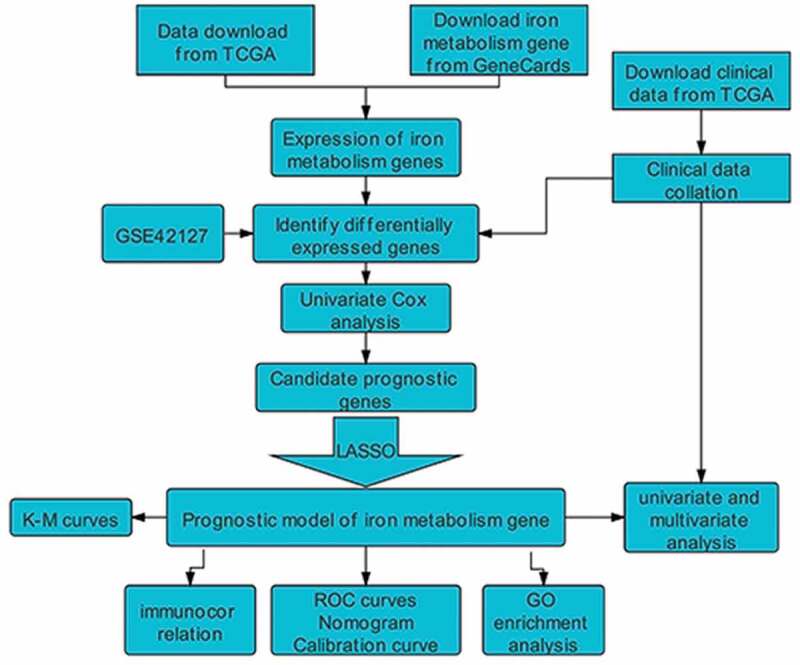

## Introduction

Lung cancer is the leading cause of cancer-related deaths around the world [1]. In 2018, there were 2.1 million new lung cancer cases and 1.8 million deaths worldwide [2]. In recent years, the incidence of lung adenocarcinoma has consistently increased and has caused it to become the most common type of non-small cell lung cancer [3]. Thus, it is necessary to establish a neo-model for predicting the prognosis of lung adenocarcinoma in order to develop more effective diagnosis and treatment strategies.

Iron (Fe) is an essential nutrient for the human body; iron plays a prominent role in multiple forms of cell death, including apoptosis, necrosis, ferroptosis, and ascorbate-mediated death [4]. Circulating iron is normally found complexed with transferrin (Tf) and circulates in the bloodstream. Tf is absorbed in peripheral tissues by binding to TfR1 [5]. The high expression of TfR1 is not only related to the reduced response to chemotherapy, but also to the increased phosphorylation of Src kinases in breast cancer, promoting tumor cell division, motility and adhesion [5]. Disorders of iron metabolism in cancer are well known. Based on review of the literature, there is evidence that iron plays a particularly important role in lung cancer [6]. Disorders of iron metabolism are closely linked to the occurrence, proliferation and progression of tumors, and seriously affect tumorigenesis [7]. Sukiennicki [8] et al. showed that high iron and high iron protein represent higher body iron, which may be relevant to the occurrence of lung cancer. Ferritin and SOD are widely recognized in the occurrence of lung cancer [9,10]. Researchers have demonstrated that the increase of these two markers in lung cancer patients seems to be the result of inflammation and oxidative stress, and it is believed that inflammation and oxidative stress are important components of the pathogenesis of lung malignancies [10–12]. Chanvorachote [13] et al. found that iron can induce cancer stem cells and promote the production of an aggressive phenotype through the generation of ROS in lung cancer cells, which contributes to the occurrence of lung tumors. Although lung cancer is certainly not just an iron disease, these findings indicate that there is a clear and direct connection between iron and lung cancer. Therefore, it is necessary to identify novel prognostic biomarkers and construct more accurate prognostic models. Doing so can provide an effective reference for precise clinical treatment strategies for lung adenocarcinoma.

In our study, the mRNA expression profile and corresponding information data of patients with lung adenocarcinoma were obtained from the TCGA and GEO databases. We aimed to establish a credible iron metabolism-related prognostic gene signature for patients with lung adenocarcinoma. Our results help predict the prognosis of LUAD patients and provide a novel direction for the development of precise treatment strategies.

## Materials and methods

### Data collection

The Cancer Genome Atlas (TCGA) data mining platform was searched and standardized RNA-seq data was downloaded. The number of fragments per million bases (FPKM) and relevant clinical data of LUAD, were accessed and naturalized into an expression matrix [14] (As of 16 July 2020, https://portal.gdc.cancer.gov/repository). The samples consisted of mainly 594 cases of LUAD (535 samples, 59 adjacent normal samples). For the clinical information materials of TCGA-LUAD patients, the following methods were used for preprocessing: (1) Samples without clinical data were deleted; (2) Samples with follow-up time lower than 30 days were deleted. In total, 486 LUAD patients were included in the research as a training set.

Iron metabolism genes were downloaded through the GeneCards data portal, screening out the relevant score threshold (relevant score ≥ 5), and finally 3037 iron metabolism-related genes were obtained (https://www.genecards.org/). The mRNA expression matrix of iron metabolism genes in this study was obtained by taking the intersection with the expression matrix of the above-mentioned TCGA-LUAD patients, which was then used for subsequent analysis.

In addition, we retrieved gene expression arrays (GSE42127) and clinical information materials of another 133 lung adenocarcinoma patients in the Gene Expression Omnibus (GEO) (https://www.ncbi.nlm.nih.gov/geo/) in order to verify the prognostic status of the gene signatures found in the training set (TCGA). Similarly, we deleted samples with no clinical data and with a follow-up time lower than 30 days. Finally, 131 patients were used as a test set for further validation.

### Identify differentially expressed genes by dimension reduction algorithm

The ‘SVA’ R software was used to eliminate batch effects and other unnecessary changes in high-throughput experiments; the intersection genes of the TCGA and GEO data sets were obtained respectively [15]. Next, the ‘limma’ R software package was used to further distinguish the differentially expressed genes (DEGs) between the tumor tissue and the tumor adjoining tissue ((FDR)<0.05, logFC = 1.5) [16]. Finally, univariate Cox analysis was executed to single out iron metabolism-related genes with a strong prognostic ability (P < 0.01). The candidate metabolic genes obtained were used in the next step of constructing the prognostic gene signature.

### PPI network construction

The candidate genes related to iron metabolism were obtained by univariate Cox analysis. On the STRING database portal (version 11.0) [17], the protein-protein interaction (PPI) network of candidate genes was downloaded. R software package was used to compute the correlation coefficients of iron metabolism candidate genes and construct a correlation network diagram.

### Construction and evaluation of iron metabolism gene prognosis model

For the candidate genes obtained above, in order to prevent overfitting, LASSO-Cox regression analysis was carried out through the ‘glmnet’ package, and a predictive prognostic model containing 12 genes related to iron metabolism (iteration = 2000) was constructed [18]. The LASSO penalty was applied to simultaneous consideration of contraction and variable selection [18,19]. The penalty parameter (λ) of this metabolic model was confirmed through 10-fold cross-validation based on the ‘glmnet’ software package in the R software [18]. On the basis of standardized expression levels of a piece iron metabolic genes and its regression coefficient, the risk score equation of LUAD patients was calculated as follows:

Risk Score = ∑ (The expression level of a piece metabolic gene × regression coefficient).

Patients with lung adenocarcinoma could be split into high-risk and low-risk groups, on the basis of the median risk score. Kaplan-Meier analysis was carried out and the ‘survival ROC’ software package was applied in order to draw time-dependent ROC curves [20,21]. In addition, it was possible to assess the predictive performance of the metabolic gene signature using a calibration chart that compared, predicted, and observed overall survival (OS) [20]. The GSE42127 data set with clinical data was used for further external verification.

### Prognostic independence analysis of lung adenocarcinoma

There was a need to further determine independent prognostic parameters and verify the powerful prognostic ability of gene signature. Therefore, in order to conduct the study of gene signature and clinical pathological parameters (mainly age, gender, stage, TNM stage) that predict prognosis in the TCGA data set, we conducted univariate and multiple Cox regression analysis. Among them, in the multivariate Cox regression analysis, P < 0.05 was considered statistically significant. Therefore, we only considered the parameters with P value <0.05 in the univariate analysis.

### Potential correlations between high and low risk populations and biological functions and immune cells

To explore the molecular mechanisms of the metabolic gene signature, we executed gene set enrichment analysis (GSEA) to further validate the model (version GSEA_4.0.3) [22]. Before that, we divided LUAD patients into high-risk and low-risk groups. Then, based on the gene expression data of lung adenocarcinoma patients obtained from TCGA, the ESTIMATE (using gene expression profile to assess stromal cells and immune cells in malignant tumor tissues) algorithm was used to calculate stromal, immune and estimated scores [23]. Subsequently, using the ‘gsva’ software package in the R software, we performed a single-sample gene set enrichment analysis (ssGSEA) [24]. Through the application and estimation of expression data, valuable insights into the state of immune cell infiltration and the activity of immune-related pathways were obtained [24].

### Statistical analysis

Statistical analysis was performed in R software v. 4.0.2. The Student’s t test was applied to analyze paired samples of tumor tissue and adjacent tumor tissue. For OS between people in different risk groups, Kaplan-Meier analysis and comparisons were applied. Through univariate and multivariate Cox regression analysis, survival assessment was further carried out. For further verification and evaluation, time-dependent ROC curve and calibration curves were drawn. Additionally, the hazard ratio (HR) and 95% confidence interval (CI) was computed. The stromal, immune and estimated scores were calculated using the ESTIMATE software package. In all the statistical tests involved in this research, a P value < 0.05 was considered statistically significant.

## Results

In the present study, we aimed to identify genes involved in iron metabolism that affect the prognosis of LUAD. We identified 12 DEGs involved in iron metabolism. These DEGs were used to construct a new prognostic models and validate it to explore the prognostic predictive power and diagnostic power of the signature. In addition, we also performed functional enrichment analysis and immune correlation analysis to explore the potential biosynthetic mechanisms involved in the pathogenesis of lung adenocarcinoma.

### Identification of iron metabolism DEGs in LUAD

**Figure 1.**
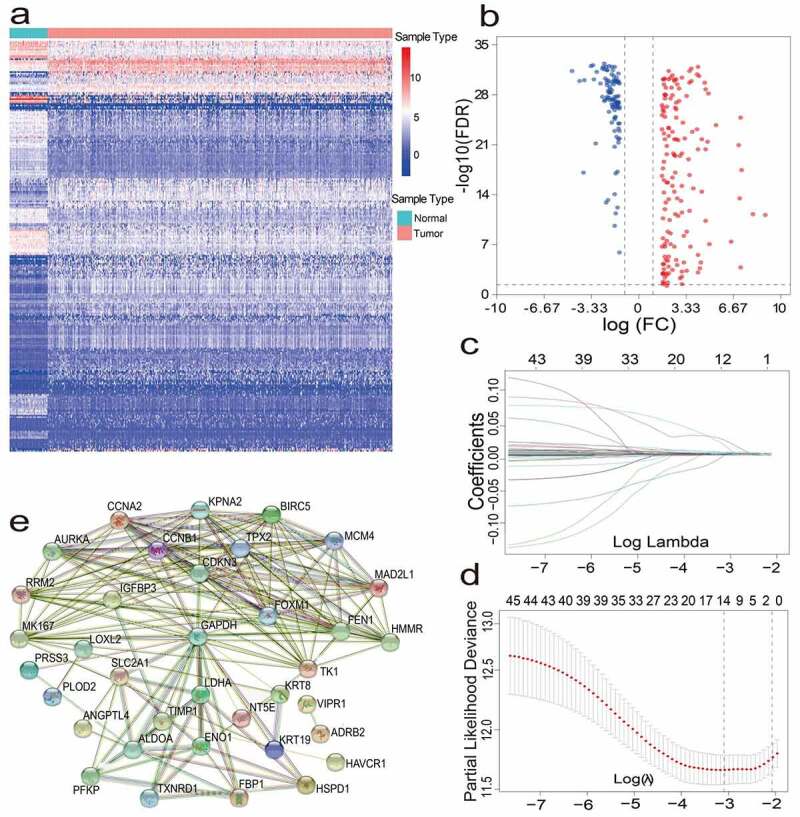
(a)(b) Heat map and volcano map of 257 different gene expression levels. (c) Coefficient distribution of 12 prognostic genes. (d) The dashed lines represent the minimum value and the optimal λ of the optimal volume of the variable respectively. (e) PPI network downloaded from STRING database shows the interaction among 46 candidate genes. Correlation coefficients are expressed in different colors

After pre-processing, 486 LUAD patients from TCGA and 131 lung adenocarcinoma patients from GEO were selected. A detailed summary of the clinical features of these patients is shown in Table 1. In order to identify prognostic genes related to iron metabolism of LUAD, differential expression analysis was performed. The DEGs between tumor samples and neighboring tumor samples were selected through the Wilcox Test. A total of 257 iron metabolism-related DEGs were identified (adjusted p values<0.05 and |logFC|>1.5); among them, there were 154 up-regulated DEGs, and 103 significantly down-regulated DEGs. The heat map and volcano map of these differential genes are shown in (Figure 1(a,b)). To further identify the representative prognostic genes of iron metabolism, we performed univariate Cox analysis, leading to the retention of 46 DEGs (P < 0.01, Table 2). The interaction network between these genes is shown in (Figure 1(e)).

**Table 1.** Clinical characteristics of the lung cancer patients used in this study

Features	TCGA (n, %)	GSE42127 (n, %)
Platform	Illumina HiSeq	Illumina HumanWG-6 v3 Array
≤ 60 years	156 (32.1%)	22 (16.8%)
> 60 years	330 (67.9%)	109 (83.2%)
NA	0 (0.0%)	0 (0.0%)
Male	225 (46.3%)	67(51.1%)
Female	261 (53.7%)	64 (48.9%)
NA	0 (0.0%)	0 (0.0%)
StageI	258 (53.1%)	87(66.4%)
StageII	116 (23.9%)	22(16.8%)
StageIII	79 (16.3%)	20(15.3%)
StageIV	25 (5.1%)	1 (0.75%)
NA	8 (1.6%)	1 (0.75%)
T1	163 (33.5%)	123 (93.9%)
T2	259 (53.3%)	7 (5.35%)
T3	43 (8.9%)	0 (0.0%)
T4	18 (3.7%)	0 (0.0%)
TX	3 (0.6%)	1(0.75%)
NA	0(0.0%)	0(0.0%)
N0	312 (64.2%)	一
N1	93 (19.1%)	一
N2	68 (14.0%)	一
N3	2(0.4%)	一
N4	0(0.0%)	一
NX	10 (2.1%)	一
NA	1(0.2%)	一
M0	322 (66.3%)	一
M1	24 (4.9%)	一
MX	136 (28.0%)	一
NA	4 (0.8%)	一
Alive	328 (67.5%)	90 (68.7%)
Dead	158 (32.5%)	41 (31.3%)

**Table 2.** Univariate Cox analysis results of TCGA cohort-46 candidate genes

Gene	HR	95% CI(low)	95% CI(high)	P value
AURKA	1.025	1.008	1.042	0.003
FBP1	0.993	0.988	0.998	0.004
MKI67	1.045	1.021	1.069	<0.001
CYP4B1	0.994	0.989	0.998	0.005
HAVCR1	1.133	1.071	1.199	<0.001
FEN1	1.028	1.011	1.046	0.001
CYP27A1	0.984	0.973	0.996	0.007
MCM4	1.019	1.006	1.033	0.003
RRM2	1.026	1.012	1.04	<0.001
ITGB4	1.006	1.002	1.01	0.004
VIPR1	0.839	0.753	0.936	0.002
ENO1	1.002	1.001	1.002	<0.001
INHA	1.008	1.003	1.014	0.004
HSPD1	1.006	1.003	1.009	<0.001
ADRB2	0.785	0.673	0.914	0.002
PFKP	1.009	1.004	1.013	<0.001
TK1	1.009	1.003	1.014	0.002
CCNB1	1.019	1.009	1.029	<0.001
TXNRD1	1.002	1.001	1.004	0.001
PLOD2	1.013	1.007	1.02	<0.001
MAD2L1	1.05	1.012	1.09	0.009
SPN	0.896	0.831	0.966	0.004
BIRC5	1.023	1.008	1.038	0.003
KRT19	1.001	1	1.001	0.004
GAPDH	1.001	1	1.001	<0.001
KPNA2	1.012	1.006	1.017	<0.001
ANGPTL4	1.009	1.004	1.013	<0.001
CCNA2	1.034	1.016	1.052	<0.001
PRSS3	1.024	1.009	1.04	0.002
KRT8	1.001	1.001	1.002	<0.001
LDHA	1.005	1.003	1.006	<0.001
HMMR	1.075	1.041	1.11	<0.001
ABCC2	1.019	1.007	1.031	0.001
CDKN3	1.039	1.013	1.066	0.003
SLC2A1	1.01	1.007	1.012	<0.001
FOXM1	1.035	1.017	1.053	<0.001
SCN4B	0.763	0.63	0.923	0.005
NT5E	1.009	1.002	1.016	0.008
DLC1	0.96	0.932	0.99	0.009
IGFBP3	1.003	1.001	1.004	0.002
CYP24A1	1.003	1.002	1.005	<0.001
LOXL2	1.02	1.014	1.027	<0.001
TIMP1	1.001	1	1.002	0.001
ALDOA	1.002	1.001	1.003	0.002
PTPRH	1.036	1.012	1.061	0.003
TPX2	1.011	1.004	1.019	0.001

### Establishment and verification of an iron metabolism-related gene signature

**Figure 2.**
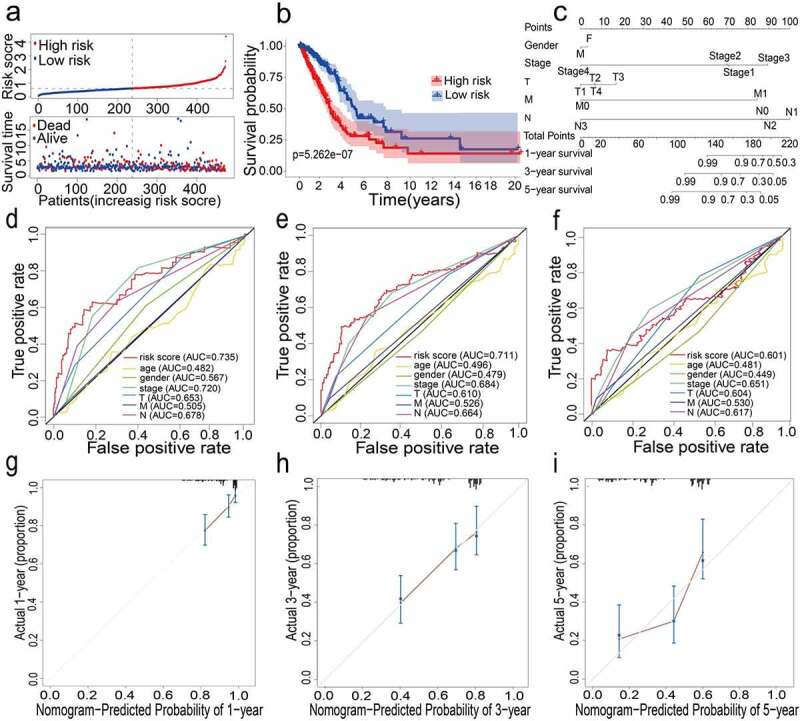
(a) Distribution of median of risk scores and OS status and risk score in TCGA cohort. (b) Survival analysis of TCGA high-risk group and low-risk group (P < 0.001). (c) Nomogram analysis results of TCGA cohort. (d)(e)(f) AUC of time-dependent ROC curves in TCGA cohort for 1 year, 3 years and 5 years. (g)(h)(i) Calibration curve for 1 year, 3 years and 5 years in TCGA cohort

For constructing a genetic signature related to iron metabolism, the following steps were performed: first, on the basis of expression profiles of the above 46 candidate genes, LASSO Cox regression analysis (2,000 iterations) was carried out. According to the minimum λ, the optimal model was constructed with the minimum parameters (Figure 1(c,d)). Eventually, a prognostic model containing 12 genes was established to evaluate the prognosis of each lung adenocarcinoma patient. The specific calculation equation for this risk score was: Risk Score = (0.01949× expression value of HAVCR1 －0.00501 × expression value of SPN + 0.00003× expression value of GAPDH + 0.00087 × expression value of ANGPTL4 + 0.00004× expression value of PRSS3 + 0.00036 × expression value of KRT8 + 0.00122 × expression value of LDHA + 0.02521× expression value of HMMR + 0.00407 × expression value of SLC2A1 + 0.00102× expression value of CYP24A1 + 0.00450 × expression value of LOXL2 + 0.00031 × expression value of TIMP1). Patients could be split into high-risk group (n = 238) and low-risk group (n = 239) using the optimal cutoff value of the risk score (After adjusted, Figure 2(a)). Kaplan-Meier analysis results revealed that the OS of the two different risk groups in the training group was significantly different. It was observed that the OS of the low-risk group was significantly higher than that of the high-risk group (P < 0.0001, Figure 2(b)). Next, the strong prognostic value of 12 gene signatures was analyzed using the time-dependent ROC curve. In addition, with respect to the prediction of risk scores for 1-year, 3-year, and 5-year overall survival, the AUCs were 0.735, 0.711, and 0.601, respectively (Figure 2(d,e,f)).

### External verification of 12 gene signatures in GSE42127

**Figure 3.**
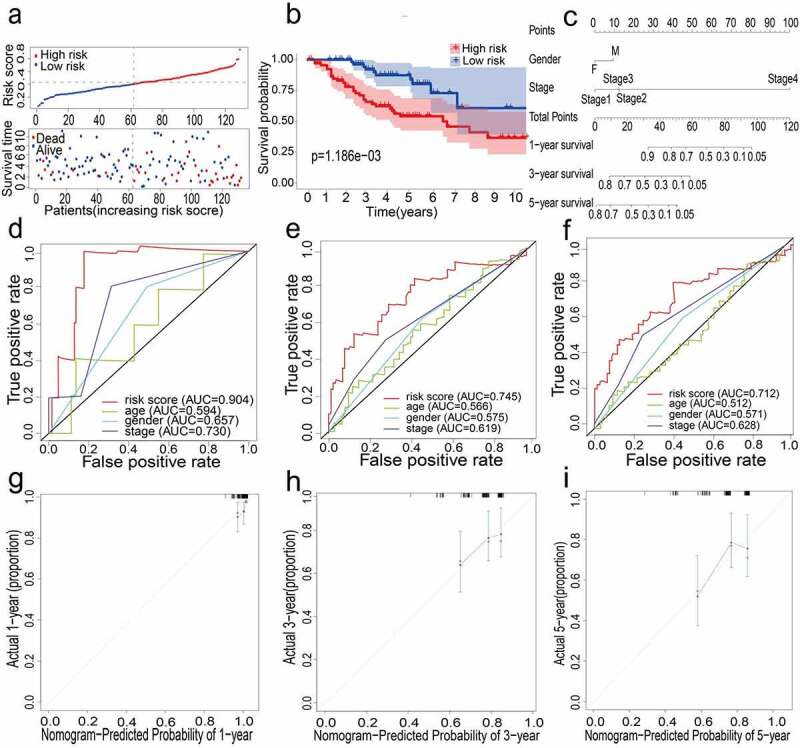
(a) Distribution of median of risk scores and OS status and risk score in GEO cohort. (b) Survival analysis of GEO high-risk group and low-risk group (P = 0.001). (c) Nomogram analysis results of GEO cohort. (d)(e)(f) AUC of time-dependent ROC curves in GEO cohort for 1 year, 3 years and 5 years. (g)(h)(i) Calibration curve for 1 year, 3 years and 5 years in GEO cohort

The external data set GSE42127 further proved the predictive capability of the 12-gene prognostic signature. For patients in the GEO cohort, the same calculation method as the TCGA cohort was applied to compute the risk score, following which the LUAD patients were split into high-risk and low-risk groups (Figure 3(a)). Kaplan-Meier analysis results were similar to those obtained in the TCGA cohort; it was shown that the overall survival of the low-risk group was significantly longer than that of the high-risk group (P = 0.001, Figure 3(b)). Next, the prognostic ability of the signature was assessed through time-dependent ROC, wherein the 12-gene signature could have a higher performance. When predicting the AUC of the overall survival (OS) of the 12-gene signature, the results at 1, 3 and 5 years were 0.904, 0.745, and 0.712, respectively (Figure 3(d,e,f)).

### Analysis of independent prognostic potency of 12-gene signature

To determine the prognostic factors of overall lung adenocarcinoma survival, we carried out univariate and multivariate Cox regression analysis. Among them, the univariate analysis showed the following results for the TCGA cohort: Risk Score (HR = 3.982, 95%CI = 2.867–5.530, P < 0.001), Stage (HR = 1.648, 95%CI = 1.396–1.946, P < 0.001), T stage (HR = 1.600, 95%CI = 1.285–1.994, P < 0.001), N stage (HR = 1.787, 95%CI = 1.455–2.195, P < 0.001). The univariate analysis also showed that the GEO cohort with Risk Score (HR = 82.970, 95%CI = 10.025–686.710, P < 0.001), Stage (HR = 1.652, 95%CI = 1.144–2.387, P = 0.007) had a significant correlation with the overall survival of lung adenocarcinoma (Table 3). Interestingly, we observed that the risk scores in the TCGA and GEO cohorts were distinctly related to OS. Similarly, the multivariate regression analysis (after correcting the parameters) indicated the following data for the TCGA cohort: Risk Score (HR = 3.313, 95%CI = 2.273 − 4.827, P < 0.001), Stage (HR = 1.921, 95%CI = 1.154–3.198, P = 0.012); and the GEO cohort: Risk Score (HR = 84.063, 95%CI = 7.882 − 896.052, P < 0.001), Stage (HR = 1.568, 95%CI = 1.052–2.337, P = 0.027) (Table 3). However, in the multivariate Cox regression analysis, the risk score was an independent predictor of OS.

**Table 3.** Univariate and multivariate Cox analysis of the 12-gene prognostic signature and clinical risk factors

Variables	Univariate analysis			Multivariate analysis		
**Training set**						
**id**	**HR**	**HR (95% CI)**	**p-value**	**HR**	**HR (95% CI)**	**p-value**
Age	0.997	0.978–1.015	0.718	1.014	0.994 − 1.035	0.16
Gender	1	0.694–1.441	1	0.85	0.585 − 1.235	0.394
Stage	1.648	1.396–1.946	<0.001	1.921	1.154 − 3.198	0.012
T	1.6	1.285–1.994	<0.001	1.009	0.785 − 1.296	0.946
M	1.748	0.959–3.187	0.068	0.368	0.095 − 1.423	0.147
N	1.787	1.455–2.195	<0.001	0.943	0.603 − 1.476	0.798
Risk Score	3.982	2.867–5.530	<0.001	3.313	2.273 − 4.827	<0.001
**Test set**						
**id**	**HR**	**HR (95% CI)**	**p-value**	**HR**	**HR (95% CI)**	**p-value**
Age	1.01	0.977 − 1.044	0.561	0.985	0.950 − 1.021	0.407
Gender	1.905	0.994 − 3.650	0.052	1.23	0.616 − 2.454	0.557
Stage	1.652	1.144 − 2.387	0.007	1.568	1.052 − 2.337	0.027
RiskScore	82.97	10.025 − 686.710	<0.001	84.063	7.882 − 896.502	<0.001

HR: Hazard ratio; CI:confidence interval; T: Tumor; M: Metastasis; N: Node.

### Constructed and verified nomogram and calibration plots

All the clinical information parameters in the univariate Cox regression analysis mentioned above exist in the TCGA and GEO cohorts. Among these, gender, stage, T stage, M stage and N stage were the parameters involved in the TCGA nomogram (Figure 2(c)). The parameters included in the GEO nomogram were gender and stage (Figure 3(c)). In the TCGA and GEO cohorts, a prognostic nomograph was constructed to predict the OS at 1, 3, and 5 years, respectively. It can be seen that those patients with higher scores have distinctly lower OS than those with lower scores. In addition, the results of the calibration chart have shown that the nomogram is significantly accurate in predicting the OS of patients with lung adenocarcinoma (TCGA: Figure 2(g,h,i), GEO: Figure 3(g,h,i)).

### Biological function and immune analysis of TCGA Cohort

**Figure 4.**
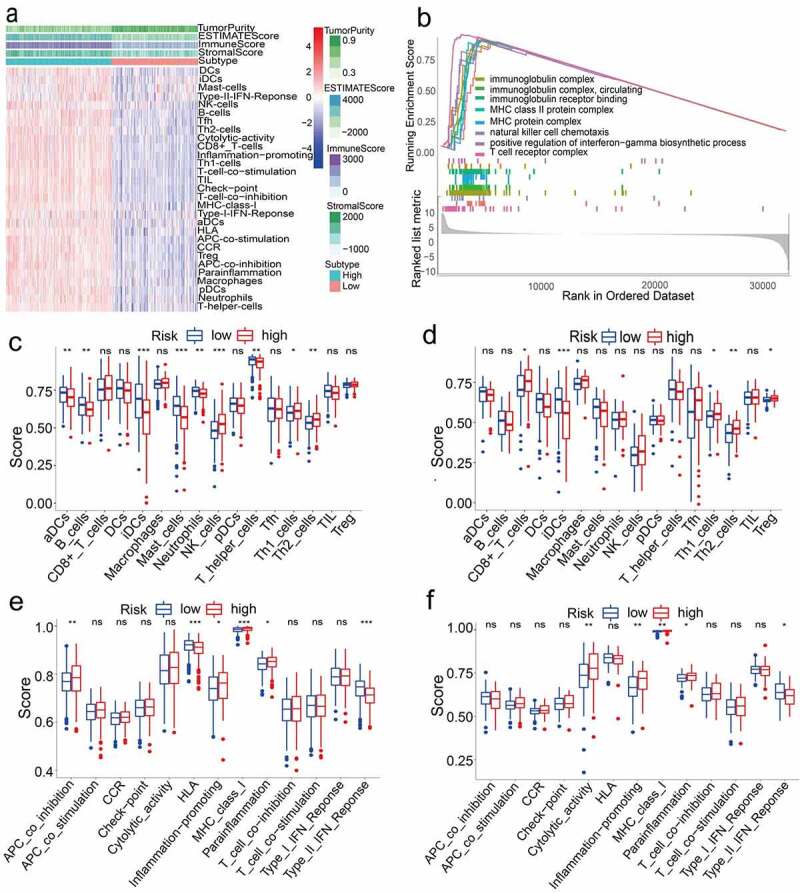
(a) Immune grouping results and tumor microenvironment heat map. Distribution of tumor purity, ESTIMATE score, immune score, and stromal score in high vs low immunity groups. (b) GO enrichment analysis results (P < 0.05). (c)(d)(e)(f) Results of immune cell scores and immune-related functions in TCGA and GEO groups

Next, we aimed to deepen our understanding of the biological functions of the prognostic model. In 477 LUAD samples from high-risk and low-risk groups of TCGA (After adjusted), ssGSEA was used to explore the tumor microenvironment in different immune clusters, and to compute the stromal score, immune score and estimated score of cancer tissue expression profile. Based on the data, we have reason to conclude that patients with high immunity have higher estimated score, stromal score, and immune score than patients with low immunity. In contrast, the tumor purity of the low-immune patients was higher than that of the high-immune patients. The result is shown in Figure 4(a). These results indicated that the TCGA cohort significantly enriched many immune-related biological processes (P < 0.05). Among them, eight biological processes related to immunity include: immunoglobulin complex, natural killer cell chemotaxis, circulating immunoglobulin complex, immunoglobulin receptor binding, MHC class II protein complex, MHC protein complex, positive regulation of interferon−gamma biosynthetic process, and T cell receptor complex (P < 0.05, Figure 4(b)).

(*** P < 0.001, ** P < 0.01, * P < 0.05, ns = not significant).

We also probed the correlation between high and low-risk populations and immune status. ssGSEA analysis was used to analyze the immune cells and related functions of the two groups. Between the low-risk group and the high-risk group of the TCGA cohort, it was observed that the scores of APC co-inhibition, Th2 cells, NK cells and MHC class I were significantly different (P < 0.05, Figure 4(c,e)). In addition, for the high-risk group, the scores of Inflammation-promoting, Parainflammation and Th1 cells were higher, while the scores were lower for HLA, type II IFN response, aDCs, B cells, iDCs, Mast cells, Neutrophils and T helper cells (P < 0.05, Figure 4(c,e)). In the ssGSEA score of the GEO cohort, between the high-risk group and the low-risk group, there were differences in cytolytic activity, MHC class I, Parainflammation, type II IFN response, Th2 cells, CD8 + T cells, iDCs, Inflammation−promotion, Th1 cells, and Treg (P < 0.05, Figure 4(d,f)).

## Discussion

Lung cancer is a malignant lesion formed by the immortal proliferation of cancer cells with genetic mutations in the lungs. Lung adenocarcinoma is the most common type of lung cancer. Studies have shown that malignant cancer is usually related to dysregulated iron metabolism, especially the expression of iron metabolism genes. This excess iron is needed not only in the early stages of tumor development, but also in the late stages of promoting the metastatic cascade [25,26]. In view of the complex network of iron metabolism genes in cancer cells and their effects on tumor growth and survival, it is necessary to understand their relevance to the prognosis of lung adenocarcinoma. In present study, we identified DEGs related to iron metabolism, and then constructed a 12-gene prognostic model through the LASSO Cox regression analysis and verified its relationship with OS in the external cohort (GSE42127). The results indicated that the 12-gene signature was able to divide LUAD patients in the TCGA and GEO datasets into two groups with different risk levels, namely the high-risk group and the low-risk group. Kaplan-Meier analysis suggested that patients with low-risk scores were correlated with better prognosis, and vice versa. These results indicated that our gene model significantly correlated with the overall survival of LUAD patients. In addition, univariate and multivariate Cox analysis results revealed that our signature model was closely related to risk scores. Risk scores are an extremely important factor that predict the prognosis of patients, which further reflects the strong prognostic ability of our signature. ROC analysis, nomogram and calibration graphs using TCGA and GEO data sets also confirmed the robustness of our prognostic model. In addition, functional and immune exploration analysis showed that immune-related pathways were enriched. Therefore, these results indicated that our 12-gene signature provides the possibility of identifying lung adenocarcinoma and using iron metabolism genes to establish a prognostic model.

The gene signature proposed in this study consists of 12 iron metabolism-related genes (HAVCR1, SPN, GAPDH, ANGPTL4, PRSS3, KRT8, LDHA, HMMR, SLC2A1, CYP24A1, LOXL2, TIMP1). Hepatitis A virus cellular receptor 1 (HAVCR-1) is mainly a susceptibility gene for asthma and allergies, which is principally expressed on Th2 cells and acts as an effective costimulatory molecule for T cell activation [27,28]. According to a report by Zheng et al. [29] the abnormal expression of HAVCR-1 is associated with the occurrence and progression of NSCLC. Glyceraldehyde-3-phosphate dehydrogenase (GAPDH) is a glycolytic enzyme and one of the main housekeeping proteins, and its increased expression is correlated with the proliferation and invasion of lung cancer [30]. Angiopoietin-like protein 4 (ANGPTL4) is a glycoprotein secreted by various cells; it belongs to the Angiopoietin family (ANGPTL) and is overexpressed in non-small cell lung cancer [31]. According to Ma et al. [32] serine protease 3 (PRSS3) and its signal transduction pathway are related to poor prognosis in lung cancer, which may lead to the invasion and growth of lung adenocarcinoma tumor cells. Keratin 8 (KRT8) is a type II basic intermediate filament (IF) protein, which can be abnormally expressed in various human cancers (including lung adenocarcinoma tissue) [33]. Lactate dehydrogenase A (LDHA) is an enzyme that plays a particularly important role in cancer cell metabolism and tumor growth, and is connected with poor prognosis in lung adenocarcinoma [34,35]. Hyaluronan-mediated motor receptor (HMMR) is a multifunctional protein, according to Song et al. [36]. HMMR is associated with the reduction of the overall survival of lung cancer patients. In addition, it can pass HCG18/miR-34a- The 5p/HMMR axis that can accelerate the progression of lung adenocarcinoma [37]. Glucose transporter 1 (GLUT1) is a pivotal protein in the pathway of cellular energy metabolism, also known as solute carrier family 2 member 1 (SLC2A1); it has a particularly essential role in the occurrence and progression of tumors, and may be one of the driver genes of lung cancer [38]. Cytochrome P450 family 24 subfamily A member 1 (CYP24A1) is situated at the inner mitochondrial membrane and nucleus, according to Shiratsuchi et al [39]. The expression of CYP24A1 is relevant to the poor prognosis of resected lung adenocarcinoma. Lysine oxidase-like 2 (LOXL2) pertains to the lysyl oxidase (LOX) family, and is mainly involved in the formation of cross-linked products of matrix collagen and elastin outside the cell [40]. In addition, according to the report by Peng et al. [41], LOXL2 has a driving effect on the invasion and metastasis of lung cancer, and the increase of LOXL2 expression indicates poor prognosis in patients with LUAD. The tumor/stroma TIMP-1 intensity ratio in the tissue has a particularly important predictive effect on tumor recurrence [42]. At present, the function of SPN in the occurrence and development of lung cancer is indistinct. Although some biological functions of these 12 genes have not been reported in LUAD, it provides a new direction for the study of tumorigenesis and cancer immunity.

In the past ten years, although iron has been a research hotspot of lung cancer, there are few studies on the correlation between iron metabolism and tumor immunity. For the patients in the different risk groups of LUAD, GO analysis was conducted. Unexpectedly, many biological processes related to immunity were enriched. Therefore, we speculate that iron metabolism may be closely related to tumor immunity. Moreover, we also studied and explored the interrelationship between risk groups and immune cells. Interestingly, there is a difference between high and low-risk groups, including naïve B cells, CD8 + T cells, activated CD4+ memory T cells, M1 Macrophages and activated dendritic cells. Previous research has indicated that CD8 + T cells [43,44] and macrophages [44,45] have a connection with the poor prognosis of lung cancer patients. Perhaps one of the reasons for the poorer prognosis of high-risk patients is the weakened anti-tumor immune function.

This study also has few deficiencies as well as limitations. First, our predictive model is constructed and verified by retrospective data from public databases. Therefore, it is necessary to conduct more prospective experimental studies to further verify the prognosis of our gene signature, and experimental studies on these genes may provide new insights into their biological functions. Secondly, the use of a single feature to build a predictive gene signature is actually an inherent defect. In practice, other mechanisms also affect the occurrence and development of lung adenocarcinoma.

## Conclusion

In summary, our research may define a new gene signature of iron metabolism to explore the overall survival of lung adenocarcinoma. The 12-gene signature consists of promising prognostic biomarkers for lung adenocarcinoma, and also provides multiple targets for precise treatment.

## Data Availability

These data were freely available in The Cancer Genome Atlas (TCGA, https://portal.gdc.cancer.gov/), Gene Expression Omnibus (GEO, https://www.ncbi.nlm.nih.gov/geo/) and Gene Cards (https://www.genecards.org/). These data are available from the corresponding author upon reasonable request. All data within the article and supplementary files are available for publish. All the raw data is publicly available.

## Abbreviations

LUAD, lung adenocarcinoma; TCGA, The Cancer Genome Atlas; GEO, Gene Expression Omnibus; Lasso: Least absolute shrinkage and selection operator; OS: overall survival; ROC: receiver operating characteristics; AUC: Area under the curve; DEGs: Differentially expressed genes; FDR: False discovery rate; GO: Gene Ontology; ssGSEA: Single-sample gene set enrichment analysis; HR: Hazard ratio; CI: Confidence interval.

## Research highlights

1. We developed the first-ever signature based on iron metabolism-related gene.

2. We uncovered a prognostic signature of 12 iron metabolism-related genes.

3.Provide a novel direction for the development of precise treatment strategies.

4. High-risk individuals showed higher tumor purity and lower immune scores.
